# Developmental genes significantly afflicted by aberrant promoter methylation and somatic mutation predict overall survival of late-stage colorectal cancer

**DOI:** 10.1038/srep18616

**Published:** 2015-12-22

**Authors:** Ning An, Xue Yang, Shujun Cheng, Guiqi Wang, Kaitai Zhang

**Affiliations:** 1 State Key Laboratory of Molecular Oncology, Department of Etiology and Carcinogenesis, Peking Union Medical College & Cancer Institute (Hospital), Chinese Academy of Medical Sciences, Beijing, 100021, China; 2 Department of Endoscopy, Cancer Hospital, Chinese Academy of Medical Sciences, Beijing, 100021, China

## Abstract

Carcinogenesis is an exceedingly complicated process, which involves multi-level dysregulations, including genomics (majorly caused by somatic mutation and copy number variation), DNA methylomics, and transcriptomics. Therefore, only looking into one molecular level of cancer is not sufficient to uncover the intricate underlying mechanisms. With the abundant resources of public available data in the Cancer Genome Atlas (TCGA) database, an integrative strategy was conducted to systematically analyze the aberrant patterns of colorectal cancer on the basis of DNA copy number, promoter methylation, somatic mutation and gene expression. In this study, paired samples in each genomic level were retrieved to identify differentially expressed genes with corresponding genetic or epigenetic dysregulations. Notably, the result of gene ontology enrichment analysis indicated that the differentially expressed genes with corresponding aberrant promoter methylation or somatic mutation were both functionally concentrated upon developmental process, suggesting the intimate association between development and carcinogenesis. Thus, by means of random walk with restart, 37 significant development-related genes were retrieved from a priori-knowledge based biological network. In five independent microarray datasets, Kaplan–Meier survival and Cox regression analyses both confirmed that the expression of these genes was significantly associated with overall survival of Stage III/IV colorectal cancer patients.

Colorectal cancer (CRC) is the third most common cancer in men (746,000 cases, 10.0% of the total) and the second in women (614,000 cases, 9.2% of the total) worldwide, accounting for roughly 694,000 deaths per year^1^. The initiation of CRC is an incredibly complicated biological process, involving multiple genomic and epigenomic alterations, occurring over an extended time period of usually a decade^2^. Patient survival is limitedly dependent on the tumor stage at the time of diagnosis, and reduced sensitivity to chemotherapy is still a major obstacle in effective treatment of advanced disease. Therefore, the discovery of novel molecules promoting CRC progression and indicating prognostic status, is still urgently needed^3^.

It is putatively accredited that carcinogenesis is caused by multi-level dysregulations, including genomics [majorly caused by somatic mutation and copy number variation (CNV)]^4^,^5^, DNA methylomics^6^,^7^, and transcriptomics^8^,^9^. CNV plays a significant role in tumorigenesis in many cancers^10^,^11^,^12^,^13^,^14^, whose accumulation during oncogenesis might be a result of preferential selection by which transforming cells gain evolutionary advantages^15^. Somatic mutation, together with CNV, could contribute to genomic instability^4^. It could also activate additional downstream pathways in many types of cancer to acquire proliferative advantages^16^,^17^,^18^. DNA methylation is substantially important in promoting embryonic development^19^, aging^20^, and nearly all types of cancer^21^,^22^,^23^,^24^, by influencing DNA and chromatin structures^25^. Numerous investigations indicated that the dysregulation of promoter region, especially promoter hypermethylation of tumor suppressor genes, was the essential epigenetic events in carcinogenesis, prognostic marker discovery, and therapeutic utilities^26^,^27^,^28^,^29^.

CNV, aberrant promoter methylation and somatic mutation could all influence gene activation or suppression, thereby influencing the process of carcinogenesis. CNVs may alter gene dosage by changing the number of copies of a gene that is present in the genome^30^,^31^,^32^,^33^, explaining in most circumstances, CNV and corresponding gene expression are positively correlated in CRC^34^. Promoter hypomethylation might lead to gene activation, and promoter hypermethylation might cause gene suppression^35^. Genes with somatic mutation could probably lead to the activation or suppression of downstream signaling pathways^36^. For example, in thyroid cancer, somatic mutation of *BRAF* could activate *MAPK* pathway, thus influencing the massive dysregulation of gene activity^37^.

The multi-level genomic dysregulations during carcinogenesis indicated that while looking into the dysregulation of gene expression in cancer, the aberrant patterns of multi-level events should also be paid considerable attention to shed light on the underlying intricate mechanisms of cancer initiation and deterioration. Therefore, the integrative analysis of cancer genomics, methylomics and transcriptomics is urgently needed to comprehensively dissect cancer etiology and provide clinical guidance.

The Cancer Genome Atlas (TCGA) database is an immeasurable source of knowledge launched in 2005, which provides publicly available cancer genomic datasets^38^. Based on abundant resources of RNA sequencing (RNAseq), DNA sequencing (DNAseq), single nucleotide polymorphism (SNP) based platforms and DNA methylation, integrative analysis of cancer genomics was exuberantly emerging, for instance, in breast cancer^39^, ovarian cancer^40^, glioma^41^, lung cancer^42^, renal cancer^43^ and many other types of cancers. Multi-dimensional analyses (MDA) of the genome, epigenome, and transcriptome was proven to be greatly beneficial in facilitating the rational deduction of aberrant genes and pathways, delineating subtypes of cancer, and promoting derivation of diagnostic and prognostic signatures, which otherwise would be overlooked in single genomic dimension investigations^44^. Thus, the molecular abnormalities of multiple levels should be altogether taken into consideration and systematically identify genes or pathways critically important in carcinogenesis.

In this study, we first collected genes with significant dysregulations with regard to DNA copy number, DNA promoter methylation, gene expression, and somatic mutation from TCGA paired samples. Differentially expressed genes (DEGs) with consistent aberrant promoter methylation or somatic mutation were found both exhibiting remarkable functional unity in developmental process. Gene to gene regulatory network was constructed by means of merging Human Protein Reference Database (HPRD), and Kyoto Encyclopedia of Genes and Genomes (KEGG) networks. By combining multi-dimensional genomic data of CRC and priori knowledge network, we applied a computational strategy, i.e. random walk with restart, to obtain the genes which were affected considerably by aberrant promoter methylation or somatic mutation. The most of these significant genes were connected in the network, and proven to hold profound prognostic information in late stage (Stage III/IV) patients, which might be helpful for constructing prognosis prediction models and providing novel tools to guide clinical implementations for this deadly disease.

## Material and Methods

**Figure 1:**
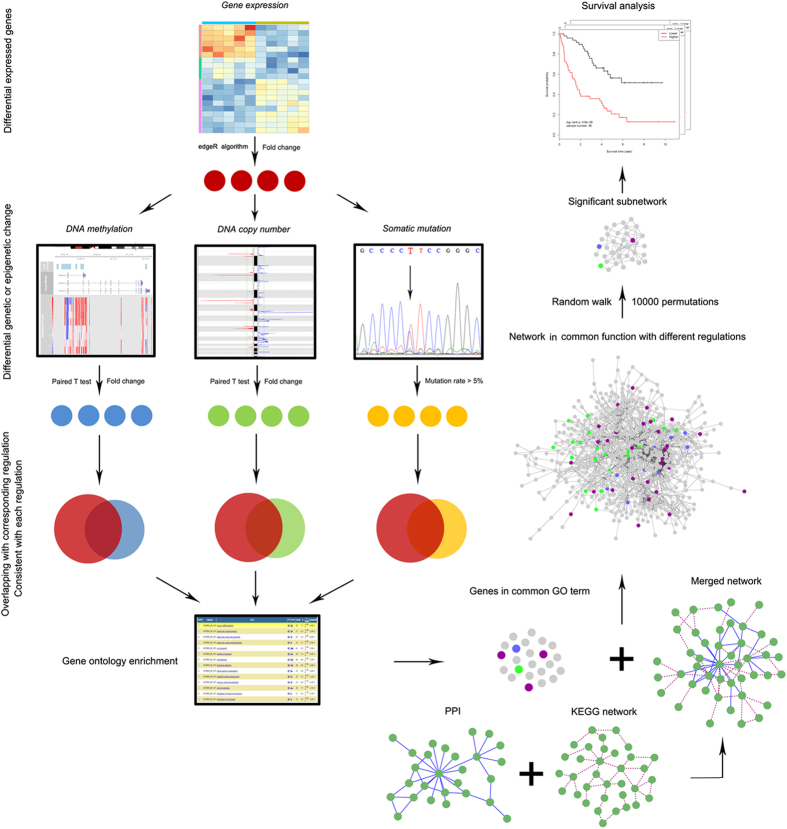
Schematic of methodology applied in this study. Step I: Integration of genomic, DNA methylomic, and transcriptomic data to identify three candidate gene groups; Step II: Identification of gene ontology (GO) function term and corresponding gene groups of interest based on GO enrichment analysis; Step III: Identification of genes within the identified functional groups significantly afflicted by genetic or epigenetic dysregulation, by applying random walk with restart algorithm in the merged network. Step IV: Survival analysis of identified significant genes to evaluate their prognostic value.

A schematic for the study is depicted in Fig. 1.

### Data retrieval

The multi-dimensional data of CRC associated datasets were retrieved from The Cancer Genome Atlas (TCGA) database (https://tcga-data.nci.nih.gov/tcga/). Four levels of paired data (cancer and normal adjacent tissues from CRC patients) were downloaded, including 32 paired RNA sequencing level 3 data [raw counts and RNASeq by Expectation Maximization (RSEM) normalized read counts], 500 paired DNA copy number level 3 data [conducted with Affymetrix SNP 6.0 platform, and segmented by circular binary segmentation (CBS) method^45^], 45 paired DNA methylation level 3 data [using Illumina HumanMethylation450 chips, and the methylation level of each CpG site was calculated as the ratio (β value) of the signal of methylated probes relative to the sum of methylated and unmethylated probes, which ranged continuously from 0 (unmethylated) to 1 (fully methylated)], and somatic mutation level 2 data of 300 patients (mutation information of 17,427 genes).

The raw data for five human CRC mRNA microarray studies with overall survival (OS) information (sample size >60, referred to as Clinicinfo superset; Table 1) were downloaded from the National Center for Biotechnology Information Gene Expression Omnibus (GEO). The flowchart of Clinicinfo dataset retrieval is presented in Supplementary Figure S1. The combined data set contained a total of 940 samples (936 samples with clear OS information) hybridized to probe sets present on both the Affymetrix HG-U133A (with GEO accession number GPL96) and the HG-U133A Plus2 (GPL570) platforms, composed of data sets with accession numbers GSE39582, GSE17536, GSE29621, GSE39084, and GSE12945. In total, 22,277 probes were common in all data sets, and of which the expression values were retrieved via robust multi-array average (RMA) algorithm and further quantile normalized using the “affy” Bioconductor package. The ComBat algorithm was utilized to eliminate potential batch effects, and the expression levels of 12,500 genes were obtained as the median value of all the probes which could be mapped to this gene. All clinical information was extracted from the original publications.

### Circos plot of TCGA colorectal data in terms of DNA copy number, DNA methylation and somatic mutation

**Figure 2:**
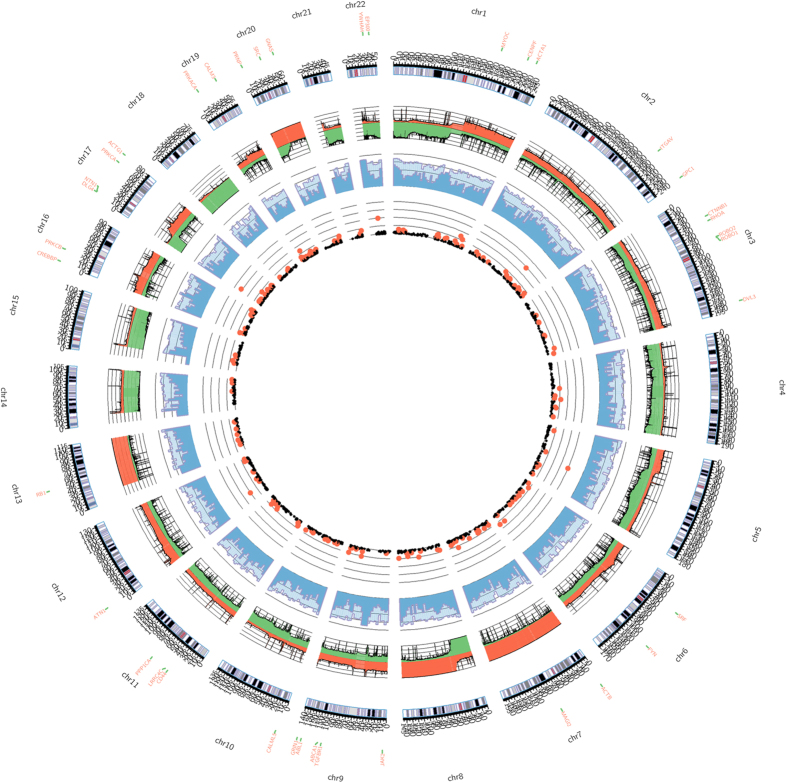
Circos plot in terms of DNA copy number, DNA methylation and somatic mutation. An ideogram of a normal karyotype is shown in the outermost ring. The next outermost ring represents DNA copy number at corresponding genomic coordinates, calculated by **t**he segment gain or loss (SGOL) scores (red represents amplification and green represents deletion). The next ring represents the amount of DNA methylation. The whole genome was segmented into contiguous 500,000 base pair (bp) bins, and the median (dark blue) and 75th percentile (light blue) of methylation levels of CpGs which could be mapped onto each bin were plotted. The innermost ring is scatter plot illustrating somatic mutation data, genes with mutation rate >5% were shown in black and those >10% were shown in red.

Colorectal primary tumor datasets in TCGA database, including 617 DNA copy number data, 393 DNA methylation data, and 300 somatic mutation data, were enrolled for integrative Circos plot construction via Perl software “Circos plot” (Fig. 2). Bioconductor package “cghMCR” was used to compute the segment gain or loss (SGOL) scores to quantify chromosome regions showing common gains/losses by summation of the score in each patient. For DNA methylation, the whole genome was segmented into contiguous 500,000 base pair (bp) bins, and the median and 75th percentile of methylation levels of CpGs which could be mapped onto each bin were plotted. As for somatic mutation data, genes with mutation rate >5% were shown in scatter plot.

### Identification of candidate genes with significant alteration at multi-level

DEGs were identified using edgeR algorithm^46^ with RNA sequencing raw counts (FDR < 0.01, fold change >2). As for DNA copy number data, Bioconductor package “CNTools” was used to process segmentation data and format the data into a gene-level matrix based on corresponding genomic location of 26,863 genes. Genes with genomic amplification and deletion were identified with paired *t* statistic test (FDR < 0.001, fold change >1.2). In methylation analysis, promoter region was defined as the region between 1,000 bp upstream transcription start site (TSS) and 300 bp downstream TSS. The β value of the probe which could be mapped to the CpG site located in the promoter region of a given gene was used to quantify the methylation level of this gene. If more than one probe could be mapped to the promoter region of a given gene, the mean value was adopted. In this manner, the methylation level of 16,996 genes were obtained with DNA methylation data, and significant hypermethylated and hypomethylated genes were identified with paired *t* statistic test (FDR < 0.001, fold change >1.5).

By virtue of dysregulation pattern at different levels, three groups of candidate genes of interest were collected: (i) genes with differential expression and corresponding copy number alteration (i.e. genes with overexpression and amplification, and genes with underexpression and deletion); (ii) genes with differential expression and corresponding promoter methylation (i.e. genes with overexpression and promoter hypomethylation, and genes with underexpression and promoter hypermethylation); and (iii) genes with differential expression and somatic mutation.

### Identification of significant genes through random walk

Gene ontology (GO) enrichment analysis was conducted using Bioconductor package “clusterProfiler”. The protein–protein interaction network was downloaded from HPRD database, and KEGG network was constructed with Bioconductor package “KEGGgraph”. Therefore, gene regulatory network was established by merging HPRD and KEGG network, including 10,479 nodes and 60,689 edges after eliminating self-loops and duplicated edges.

Taking advantage of knowledge-based network topology, random walk algorithm was utilized to identify genes algorithmically most affected by aberrant promoter methylation and somatic mutation^47^. In the network, genes of interest were designated as information source (i.e., source nodes) and the remaining genes in the network as the information target (i.e., target nodes). The information flow originates from source nodes iteratively and randomly transmits to their neighbors with a probability proportional to their topological features. At each step, the information can flow back to the source nodes with the same probability. The final steady-state probability assigned to each gene in the network reflects the integrated influence imposed by source nodes combining network topology. Formally, the random walk with restart is defined as:


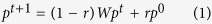


where *W* is the column-normalized adjacency matrix of network, and *p*^*t*^ is a vector in which the genes in the network holds probability in the iterative process up to step *t*. Source nodes were weighted with initial probability vector *p*^*0*^ (the sum of its elements was equal to 1), and *r* represents restart probability (*r* = 0.7 in this study). All the genes in the network were ranked according to the values in the steady-state probability vector *p*^∞^. This was obtained at query time by performing the iteration until the difference between *p*^*t*^ and *p*^*t* + *1*^ (measured by the L1 norm) was lower than 10^−10^. In order to obtain genes with significantly high steady-state probability, 10,000 permutations of node labels (with network topology remained the same) were conducted to calculate the null distribution of final probability for each gene. The *p* value was termed as the ratio of random values that were greater than the observed final probability. Genes with *p*** **<** **0.01 were regarded as the genes significantly afflicted by these genetic or epigenetic abnormalities.

### Validation of gene signature’s prognostic value in Clinicinfo superset

In order to assess the prognostic value of the significant genes we obtained (suppose the signature contained n genes), the risk score formula for predicting OS was developed based on a linear combination of the expression level (*x*_1_, *x*_2_, …, *x*_n_) of a given patient weighted by the regression coefficients derived from the Cox regression analysis. GSE17536 was used as training cohort for Cox regression model construction and the remaining four Clinicinfo data sets were treated as test cohorts. The regression coefficient *β* was calculated with training cohort and the same coefficient was further applied to testing cohorts. The risk score *r* for Patient *j* was calculated as follows:


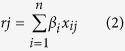


Five-fold cross validation was also conducted within training cohort to strengthen the validity of the test. We then divided patients into high-risk and low-risk groups using the median gene signature risk score. Patients with higher risk scores are expected to have significantly poor OS status, if the gene signature is closely related to OS. Kaplan–Meier survival analysis and log-rank test were performed to evaluate the prognostic difference between the two risk score assigned groups.

## Results

### Collection of genes with somatic mutation, differential expression, DNA copy number and promoter methylation with paired TCGA samples

**Figure 3:**
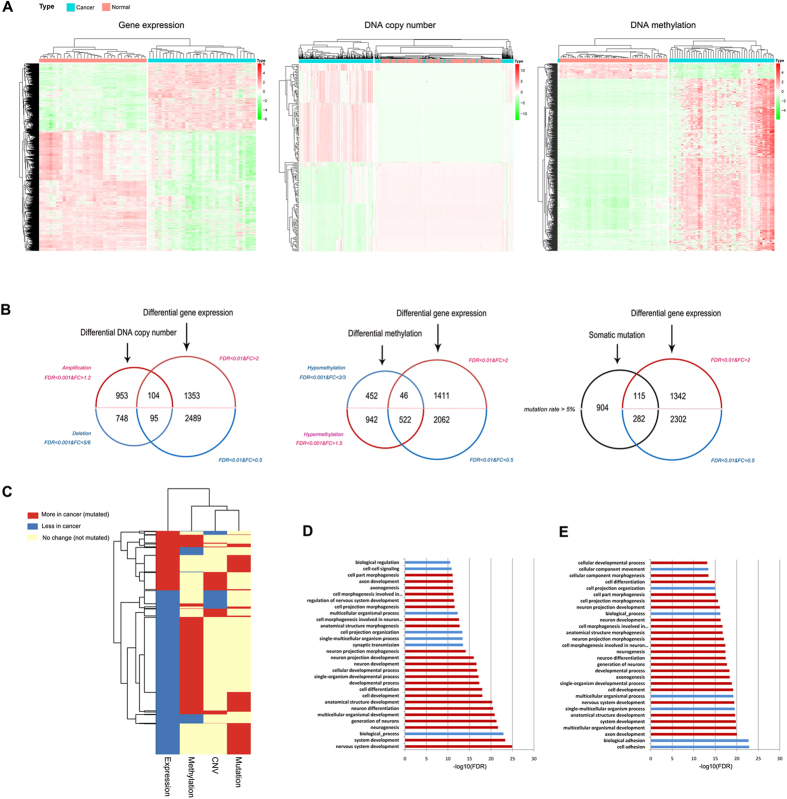
Identification of three gene groups with multi-omic data in the Cancer Genome Atlas (TCGA) database. (**a**) Heat maps of differentially expressed genes (DEGs) [log2 transformed RNASeq by Expectation Maximization (RSEM) normalized read counts], DEGs with copy number variation (CNV), and DEGs with aberrant promoter methylation in corresponding paired TCGA data, respectively. (**b**) Venn diagram illustrating three groups of candidate genes with differential expression and another altered molecular level, such as DNA copy number, promoter methylation and somatic mutation. (**c**) Integrated genetic and epigenetic alteration patterns of differentially expressed genes. Rows represent DEGs, and columns represent four dysregulation types. Red denotes the more in cancer (overexpression, promoter hypermethylation or DNA amplification) or mutated DEGs. Blue denotes less in cancer (underexpression, promoter hypomethylation or DNA deletion) or not mutated DEGs. (**d**) Gene ontology (GO) enrichment analysis of Group B genes [differentially expressed genes (DEGs) with abnormal promoter methylation]. Red bar represents enriched GO terms which are offspring of developmental process. (**e**) GO enrichment analysis of Group C genes (DEGs with somatic mutation).

Due to abundant resources of TCGA database, paired samples of CRC were used to obliterate individual difference. DEGs, calculated using edgeR algorithm, were composed of 1,457 up-regulated genes and 2,584 down-regulated genes (Fig. 3A). In addition, 1,057 genes were significantly amplified and 843 genes were found significantly deleted (Fig. 3A). Integrative Circos plot indicated there were severe copy number alteration in Chromosome 7, 8, 13, 17, 18 and 20, highly consistent with previous investigations^34^,^48^,^49^,^50^,^51^,^52^ (Fig. 2). By means of paired *t* statistic test, 1,464 genes with promoter hypermethylation and 498 genes with promoter hypomethylation were also identified (Fig. 3A), and 1,301 genes with mutation rate >5% were regarded as mutated genes.

### Identification of candidate gene groups associated with DNA copy number alterations, promoter methylation, and somatic mutation

Three groups of DEGs with aberrant genetic or epigenetic dysregulations (Fig. 3B) were categorized as follows: (i) 104 genes with overexpression and copy number amplification, and 95 genes with underexpression and copy number loss (altogether 199 genes, termed as Group A); (ii) 46 genes with overexpression and promoter hypomethylation, and 522 genes with underexpression and promoter hypermethylation (altogether 568 genes, termed as Group B); (iii) 397 genes (termed as Group C) with somatic mutation and differential expression (115 overexpression and 282 underexpression). Genetic and epigenetic dysregulation of DEGs were shown in Fig. 3C. Consistent with classic knowledge of gene regulation, promoter methylation exerted trans-regulation, while DNA copy number exerted cis-regulation upon gene expression, and the promoter of DEGs tended to be hypermethylated in CRC (Fig. 3C).

The overlapping among these three gene groups was conducted, and hypergeometric distribution was used to assess the statistical significance. The formula of hypergeometric distribution is as follows:


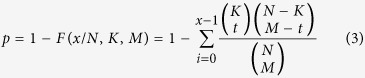


where *N* is the number of all DEGs (N = 4041, the background gene number since all candidate genes were DEGs); *K* is the gene number of one target gene groups; *M* is the gene number of the other target gene group; *x* is the number of common genes shared by the both gene groups. As shown in Supplementary Figure S2, the result of hypergeometric distribution test indicated that there was no significant overlapping between Group A and Group B (*p* = 0.966) or Group C (*p* = 0.398), while Group B significantly overlapped with Group C (n = 107, *p* = 6.309e-13).

### Random walk in developmental process related network

GO analysis of aforementioned three gene groups indicated Group A was found no GO terms significantly enriched, whereas Group B (Fig. 3D, Supplementary Table S1) and Group C (Fig. 3E, Supplementary Table S2) were both significantly enriched with a variety of GO terms (Bonferroni adjusted *FDR* < 1e-07). The enriched GO terms were increasingly ordered with FDR value, and top 30 GO terms were shown in Fig. 3D,E. All the offspring GO terms of “developmental process” were highlighted in red. Among top 30 enriched GO terms, 76.67% (23/30) of these terms were the offspring of “developmental process” for both Group B and Group C. Moreover, 48.33% (232/480, Supplementary Table S1) of Group B genes and 52.39% (186/355, Supplementary Table S2) of Group C genes belonged to this GO term (Fig. 3D,E). Among the 107 overlapping genes between Group B and Group C, 54.2% (58/107) of these genes belonged to the GO term “developmental process.

**Figure 4:**
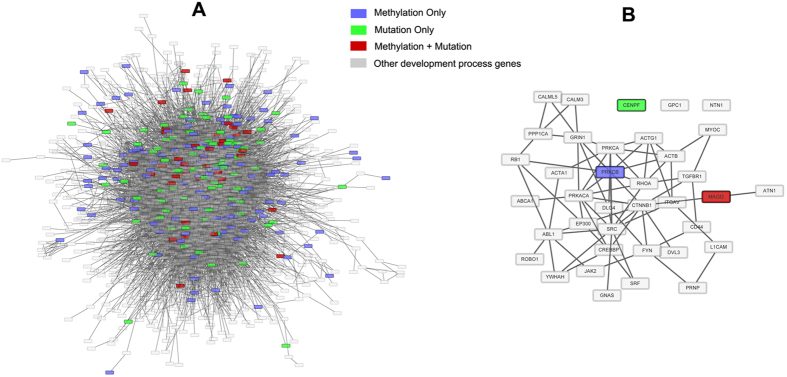
Random walk of DEGs of Group B and C in developmental process related network (DPRN). (**a**) The biggest connected component (BCC) of DPRN containing 3,271 developmental process related genes (DPRGs) and 20,652 edges. DEGs with abnormal promoter methylation and DEGs with somatic mutation in DPRN were regarded as source nodes, and the rest of DPRGs in the network were target nodes. (**b**) The subgraph of DPRN composed of 37 significant genes retrieved via random walk with restart.

Since DEGs with abnormal promoter methylation and somatic mutation were both functionally concentrated on developmental process, developmental process related genes (DPRG, n = 5,161) were extracted from GO term “GO: 0032502”. Developmental process related network (DPRN) was established by extracting DPRGs and edges between DPRGs from the aforementioned merged network. The biggest connected component (BCC) of DPRN containing 3,271 DPRGs and 20,652 edges was established as walking graph for random walk (Fig. 4A). Genes in Group B or C and also present in the BCC were used as source nodes (n = 249). Genes only afflicted with dysregulated promoter methylation or somatic mutation were scored as 1, and genes afflicted with both abnormalities were scored as 2. The initial probability vector *p*_0_ was obtained by normalizing the score vector (n = 249) so that the sum of the vector is equal to 1 (the input of random walk algorithm). When the steady-state was finally reached, all the genes in the BCC (including 249 source nodes) were scored with *p*^∞^ (n = 3271, output of random walk algorithm), and thus the genes with significantly high score were mostly affected by both of these dysregulations. Therefore, 37 significant genes in respect to steady-stage probability were collected through 10,000 permutations (Fig. 4B), and algorithmically these genes received the most influence imposed by source genes with severe genetic and epigenetic dysregulations.

### Validation of significant genes’ prognostic value via survival analysis

**Figure 5:**
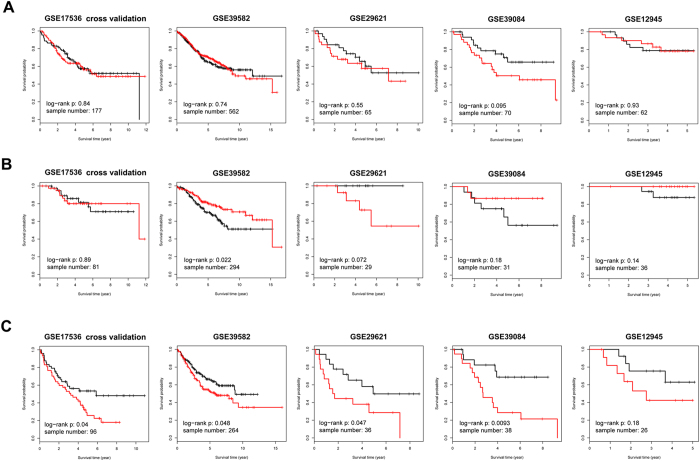
Kaplan–Meier survival analysis of random-walk significant genes in five independent datasets of Clinicinfo. (**a**) Kaplan–Meier survival analysis of 37 significant genes with all-stage samples in five independent data sets of Clinicinfo superset. GSE17536 was treated as training cohort, and five-fold cross validation was conducted to calculate risk score. Survival analysis was performed to discriminate OS between risk score assigned groups. (**b**) Kaplan–Meier survival analysis of 37 significant genes in five independent data sets with Stage I/II samples. (**c**) Kaplan–Meier survival analysis of 37 significant genes in five independent data sets with Stage III/IV samples. ***Note***: in Kaplan–Meier survival analysis, red curve represents the subgroup with higher risk score, and black curve represents lower risk score.

We used GSE17536 in Clinicinfo superset as training cohort to train Cox regression model with 37 significant genes and then used the constructed model to evaluate the risk score of patients in test cohorts. Patients in each test data set were further divided into high risk and low risk subgroups based on the median of their risk score. Kaplan–Meier survival analysis was performed to evaluate the actual survival difference between the two risk score assigned groups in samples from all American Joint Committee on Cancer (AJCC) stages (Fig. 5A), Stage I/II (Fig. 5B), and Stage III/IV (Fig. 5C) in each data set, respectively. Risk score calculated in all stage and Stage I/II samples were not significantly or consistently associated with patient’s OS in both self-cross validation and four individual test cohorts (Fig. 5A,B). However, patients with higher risk score in Stage III/IV patient groups tended to live significantly shorter than those with lower risk score. The ability of risk score to discriminate OS was quite satisfactory in Stage III/IV samples in each data set (Fig. 5C, GSE17536 cross validation, n = 96, *p* = 0.04; GSE39582, n = 264, *p* = 0.048; GSE29621, n = 36, *p* = 0.047; GSE39084, n = 38, *p* = 0.0093; GSE12945, n = 26, *p* = 0.18), suggesting the genes most influenced by promoter methylation dysregulation and somatic mutation probably hold great prognostic value in late stage CRC patients.

### Confirmation of the prognostic value of these 37 genes by means of meta-analysis and Cox regression analysis

**Figure 6:**
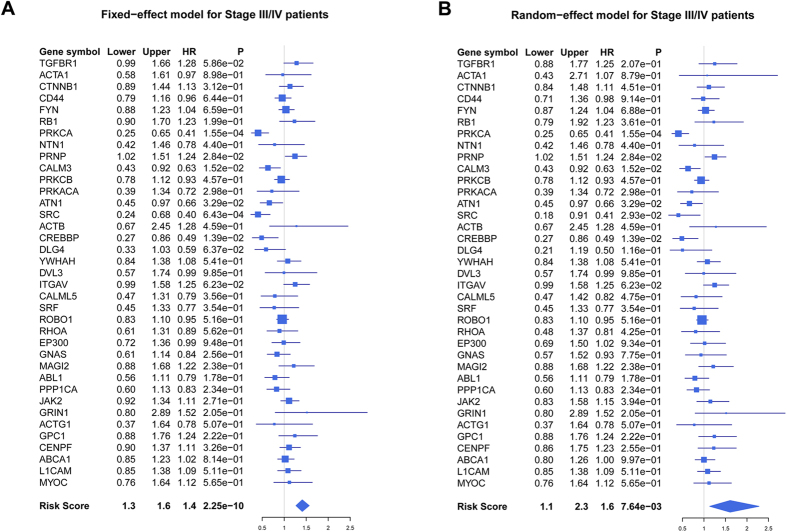
Forest plots in Stage III/IV patients in terms of overall survival (OS). (**a**) Forest plot of 37 significant genes with fixed-effect model with Stage III/IV patients in Clinicinfo superset. Meta-analysis of 37 significant genes in five independent data set of Clinicinfo superset was conducted, and hazard ratio (*HR*), 95% confidence interval (*CI*), and corresponding *p* value of each gene and risk score was calculated and plotted in the forest plot for Stage III/IV samples. (**b**) Forest plot of 37 significant genes with random-effect model.

Meta-analysis of 37 significant genes and risk score in five Clinicinfo data sets also confirmed the result of survival analysis with both fixed-effect model (Fig. 6A) and random-effect model (Fig. 6B), corroborating the prognostic value of these significant genes in late stage (conducted with R package “meta”). Fixed-effect and random-effect model are the most commonly used methods in conducting meta-analysis. The two models are different from the way of pooling the effect sizes obtained from the individual studies into an overall effect size. The fixed-effect model assumes that the differences between the studies are so important that during the effect-size pooling process, individual effect sizes should be retained; while random-effect model assumed that the individual trial effect sizes are “random” quantities^53^,^54^. Additionally, overall concordance index (C-index) analysis was also meta-analytically conducted to evaluate its OS predictive ability^55^, and the result indicated that these 37 genes could significantly predict OS of late stage CRC patients (Supplementary Fig. S3). The Cox proportional hazards regression model was used to evaluate the independence of the prognostic factors in a stepwise manner (Table 2). We collected 122 Stage III/IV samples in Clinicinfo superset with definite information of OS, age, gender, stage and grade, and univariate Cox regression analysis indicated stage [hazard ratio (*HR*): 4.384; 95% confidence interval (*CI)*: 2.671 ~ 7.194; *p* = 7.894e-09] and the risk score (*HR*: 2.225; 95% *CI*: 1.740 ~ 2.845; *p* = 4.047e-10) generated by these 37 significant genes were significantly associated with patient’s OS. Multivariate Cox analysis indicated the risk score was an independent prognostic factor (*HR*: 2.223; 95% *CI*: 1.739 ~ 2.842; *p* = 1.831e-10).

## Discussion

The booming amount of high-throughput and multi-dimensional genomic data usher us into a new era, when the tremendously complicated molecular mechanism of carcinogenesis were perceived and dissected in a more integrative perspective. In this study, we systematically analyzed CRC genomic data, including CNV, somatic mutation, DNA promoter methylation and gene expression, to discover novel and important molecules and genomic dysregulations in a more comprehensive manner. Paired samples in TCGA database were used to identify differential gene expression and genetic or epigenetic abnormalities, respectively, and collected three groups of candidate genes with differential gene expression pattern and upstream corresponding dysregulations. The result of GO analysis indicated the functions of DEGs with abnormal promoter methylation (Group B) and somatic mutation (Group C) both majorly concentrated on developmental process, of which the outcome is an anatomical structure (which may be a subcellular structure, cell, tissue, or organ), or organism over time from an initial condition to a later condition^56^. Additionally, the DEGs with CNV didn’t significantly overlap with the other DEG groups, while the majority of the significantly overlapping DEGs between Group B and Group C belonged to the GO term “developmental process” (Supplementary Fig. S2). These common DEGs shared by Group B and Group C play a pivotal role in both development and carcinogenesis. For instance, the germline gain-of-function mutation of *ALK* could disrupt the development of central nervous system^57^, of which the same anomaly was also identified in sporadic and familial neuroblastoma cases^58^,^59^,^60^,^61^. *TIAM1*, expressed in the base of intestinal crypts, established a fundamental role for *Wnt*-signaling pathway in the development and maintenance of normal intestinal physiology^62^. Its expression was greatly elevated in mouse intestinal tumors and human colon adenomas, and the cross-talk between *TIAM1* and canonical *Wnt*-signaling pathways could significantly influence intestinal tumor formation and progression^63^. Based on GO and overlapping analyses, it is quite plausible that DEGs with aberrant promoter methylation and somatic mutation intimately cooperated together to facilitate the dysregulation of developmental process. DEGs with CNV, however, were not found functionally specific in terms of influencing certain biological process.

It has been more than 150 years since Rudolf Virchow first advocated that neoplasms arise “in accordance with the same law, which regulates development” in 1858. Emerging evidences supported the cellular behavioral similarity between ontogenesis and oncogenesis, for instance, in the process of epithelial-to-mesenchymal transition (EMT)^64^, mesenchymal-to-epithelial transition (MET)^65^ and immune-surveillance evasion^66^. The molecular resemblances have been documented between certain malignant tumors and developing tissues on the basis of transcription factor activity^67^, regulation of chromatin structure^68^ and cellular signaling^69^. Important molecules were reported to play substantial role in both development and carcinogenesis. For example, *PTCH1* is a key regulator of development, whose overexpression could drive skin carcinogenesis^70^. Developmental animal models were used to uncover the complicated molecular mechanisms of carcinogenesis, and a variety of novel and pivotal molecules, pathways and biomarkers were discovered^71^,^72^,^73^. Many important signaling pathways, including *Notch1* signaling pathway, activated during development, are proven to be reactivated in the process of carcinogenesis^74^,^75^. In addition, there were some pioneering works discovering that mRNA and microRNA expression profile of cancer could recapitulate the expression pattern of development^72^,^76^,^77^,^78^,^79^. The intimate association between developmental process and carcinogenesis, together with astounding synchronization of promoter methylation dysregulation and somatic mutation in developmental process related genes (DPRGs), compelled us to propose the hypothesis that DPRGs affected most by the aberrance of promoter methylation and somatic mutation, probably hold meaningful explanation for the underlying mechanism of carcinogenesis, and might be intimately associated with clinicopathological characteristics, for instance, OS.

In our study, we adopted a simple and effective computational strategy to randomly walk DPRGs with aberrant promoter methylation or somatic mutation in HPRD and KEGG merged biological network. Random walk with restart was adopted to decipher gene to disease association in priori-knowledge based network, whose performance was proven to be much more superior to other methods, such as neighborhood approaches^80^,^81^,^82^. The advantage of this strategy is that it subtly combines observed multi-omic data with knowledge based regulatory network, tracing the information flow which would be greatly accumulated in significant genes.

The majority of these significant genes were connected to form a relatively compact biological module (Fig. 4B), implying enormous biological association existing among these genes. Many of these significant genes obtained through random walk algorithm were closely related to the initiation and progression of CRC. *TGFBR1* is a central molecule in *TGF-β* pathway, whose alteration could strikingly enhance the susceptibility to CRC^83^. The high microsatellite instability and expressional loss of *EP300* may be a feature of gastric and colorectal cancers^84^. *PRKCA* and *PRKCB* are both member of Protein kinase C (*PKC*) family, which have a role in cell proliferation, differentiation, angiogenesis, and apoptosis^85^. *PRKCB* inhibition by enzastaurin could lead to mitotic missegregation and preferential cytotoxicity toward colorectal cancer cells with chromosomal instability; loss of *PRKCA* signaling is a general characteristic of colorectal tumors regardless of other underlying genetic defects, pointing to the importance of this pathway^86^.

Since candidate genes were collected based on aberrant patterns in multi-omic level of TCGA genomic data, we used microarray data sets with OS information from GEO database instead of TCGA to test the prognostic value of these significant genes. Recent expression profiling datasets lack of consistent results between the studies due to different technological platforms and lab protocols^87^,^88^, and the microarray expression value of a particular genes could only be calculated based on different type of probes, which could probably compromise the accuracy and robustness of the whole meta-analysis. In addition, the relatively small number of sample size and noisiness of microarray data could cause the inconsistency of biological conclusions. To address these challenges, we collected five Affymetrix microarray data sets (n = 940, each sample number >60) with 22,277 common probes to get robust result of their significant clinical relevance. The expression value of 37 significant genes was retrieved and the prognostic value was evaluated with Cox regression model. The result indicated these 37 genes were significantly associated with OS in late stage (Stage III/IV) patients, rather than early stage (Stage I/II). According to AJCC staging system (7th edition)^89^, the lesion of early stage CRC (Stage I/II) is relatively contained with neither lymph node invasion nor distant metastasis; when tumor advances to late stage (Stage III/IV), the involved area is greatly increased, lymph node is invaded (Stage III/IV), and distant organs might be afflicted via distant metastasis (Stage IV). Because of the small size of tumor involvement, Stage I and Stage II patients only need to receive radical treatment to defuse the peril caused by molecularly chaotic tumors. However, with the deterioration of the disease, Stage III patients principally should be treated with neoadjuvant chemoradiation therapy followed by surgery with or without adjuvant chemotherapy, and patients with Stage IV CRC are primarily treated with chemotherapy although a selected group of patients can be cured with metastasectomy^90^. Surgical resection of the primary tumor is not beneficial for most of Stage IV patients^91^,^92^. Prognostic genes have the ability to predict patient’s OS status, probably by means of exerting influence on or reflecting tumor encroachment in the patient. Suppose the tumor is completely removed from the patient, and then the expression of this gene signature would probably not precisely predict OS, since the persistent influence of the tumor is terminated along with the tumor excision. On account of the massive tumor involvement and potential metastasis of Stage III/IV CRC, surgical excision in late stage patients might not remove the tumor with extensive molecular dysregulation as completely as in early stage patients. Therefore indicative function of prognostic genes continues monitoring the interaction between the residual neoplasms and CRC patients, probably explaining the question why these genes were only significantly associated with the OS of late stage CRC patients.

In summary, with the increasing availability of multidimensional genomic data, we collected genes with high rate of somatic mutation, differential expression, promoter methylation dysregulation and significant CNV, using paired samples in TCGA database. Three groups of DEGs with corresponding genetic or epigenetic abnormalities were obtained; the GO enrichment and overlapping analysis suggested DEGs with aberrant promoter methylation or somatic mutation were both functionally centering on developmental process. Random walk with restart was used to extract significant developmental genes most affected by aberrant promoter methylation and somatic mutation in merged regulatory network. In addition, the significant genes were closely related to OS of late stage patient. It is also very tempting that the identification of the functional regulators of these genes might be profusely beneficial to the discovery of new drug targets for CRC treatment. It is our hope that our preliminary exploration would be helpful for the further study upon cancer etiology and treatment guidance.

## Additional Information

**How to cite this article**: An, N. *et al.* Developmental genes significantly afflicted by aberrant promoter methylation and somatic mutation predict overall survival of late-stage colorectal cancer. *Sci. Rep.* **5**, 18616; doi: 10.1038/srep18616 (2015).

## Supplementary Material

Supplementary Figure S1-3

Supplementary Table S1-2

## Figures and Tables

**Table 1:** Colorectal cancer microarray datasets included in survival analysis.

Characteristics	Samples				
	GSE12945	GSE17536	GSE39582	GSE29621	GSE39084
***Number***	62	177	566	65	70
***Year***	2009	2009	2013	2014	2014
***Country***	Germany	American	France	American	France
***Gender***					
Male	34	96	310	40	35
Female	28	81	256	25	35
***Age***					
Mean ± SD (years)	64.4 ± 11.8	65.5 ± 13.1	63.0 ± 19.0	NR	59.2 ± 18.3
***T status***					
T1 + T2	16	NR	57	8	13
T3 + T4	46	NR	486	57	57
***N status***					
N0	36	NR	302	32	35
N1	14	NR	134	25	20
N2	12	NR	104	7	15
***M status***					
M0	56	NR	482	46	48
M1	5	NR	61	18	22
***AJCC stage***					
Stage I + II	36	81	297	29	31
Stage III + IV	26	96	265	36	38
***Pathologic grade***					
G I	0	16	NR	4	NR
G II	31	134	NR	51	NR
G III	31	27	NR	10	NR
***AdjCTX***					
Yes	NR	NR	233	38	NR
No	NR	NR	316	27	NR

Abbreviations: *SD* = standard deviation; *AdjCTX* = whether chemotherapy was used; *NR* = not reported.

**Table 2:** Univariate and multivariate analyses of overall survival in late stage CRC patients.

Factors	Univariate Cox regression		Multivariate Cox regression	
	HR (95% CI)	*P*	HR (95% CI)	*P*
Age	1.017 (0.998** **~** **1.037)	0.076	–	–
Gender (Male/Female)	1.245 (0.761** **~** **2.036)	0.380	–	–
Stage (IV/III)	4.384 (2.671** **~** **7.194)	**7.894e-09**	4.709 (2.839** **~** **7.812)	**1.960e-09**
Grade (III/I + II)	1.620 (0.974** **~** **2.696)	0.071	–	–
Risk score	2.225 (1.740** **~** **2.845)	**4.047e-10**	2.223 (1.739** **~** **2.842)	**1.831e-10**

Abbreviations: *HR* = hazard ratio; *CI* = confidence interval. Note: Significant *P* values were in bold (*P*** **<** **0.05).
