# Supplementary material for: Developmental genes significantly afflicted by aberrant promoter methylation and somatic mutation predict overall survival of late-stage colorectal cancer
Source: Sci Rep. 2015 Dec 22;5:18616. doi: 10.1038/srep18616 (PMC4686889; doi:10.1038/srep18616)
Supplement: Supplementary Figure S1-3 [file srep18616-s1.pdf]

Developmental genes significantly afflicted by aberrant promoter methylation and somatic mutation predict overall survival of late-stage colorectal cancer

Ning An[1,*], Xue Yang[1,*], Shujun Cheng[1,†], Guiqi Wang[2,†], and Kaitai Zhang[1,†]

[1] State Key Laboratory of Molecular Oncology, Department of Etiology and Carcinogenesis, Peking Union Medical College & Cancer Institute (Hospital), Chinese Academy of Medical Sciences, Beijing, 100021, China

[2] Department of Endoscopy, Cancer Hospital, Chinese Academy of Medical Sciences, Beijing, 100021, China

[*] These authors contributed equally to this work.

[†] Correspondence and requests for materials should be addressed to G.W. (wangguiq@126.com), K.Z. (zhangkt_bingyin@sina.cn) or S.C. (chengshj@263.net.cn)

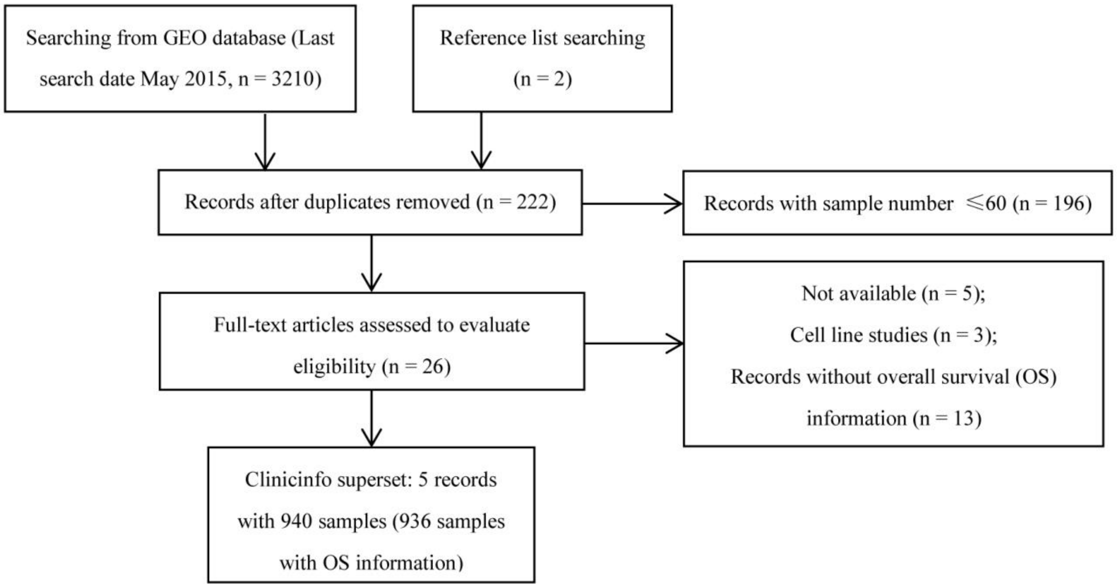

Supplementary Figure S1. Schematic of Clinicinfo dataset collection.

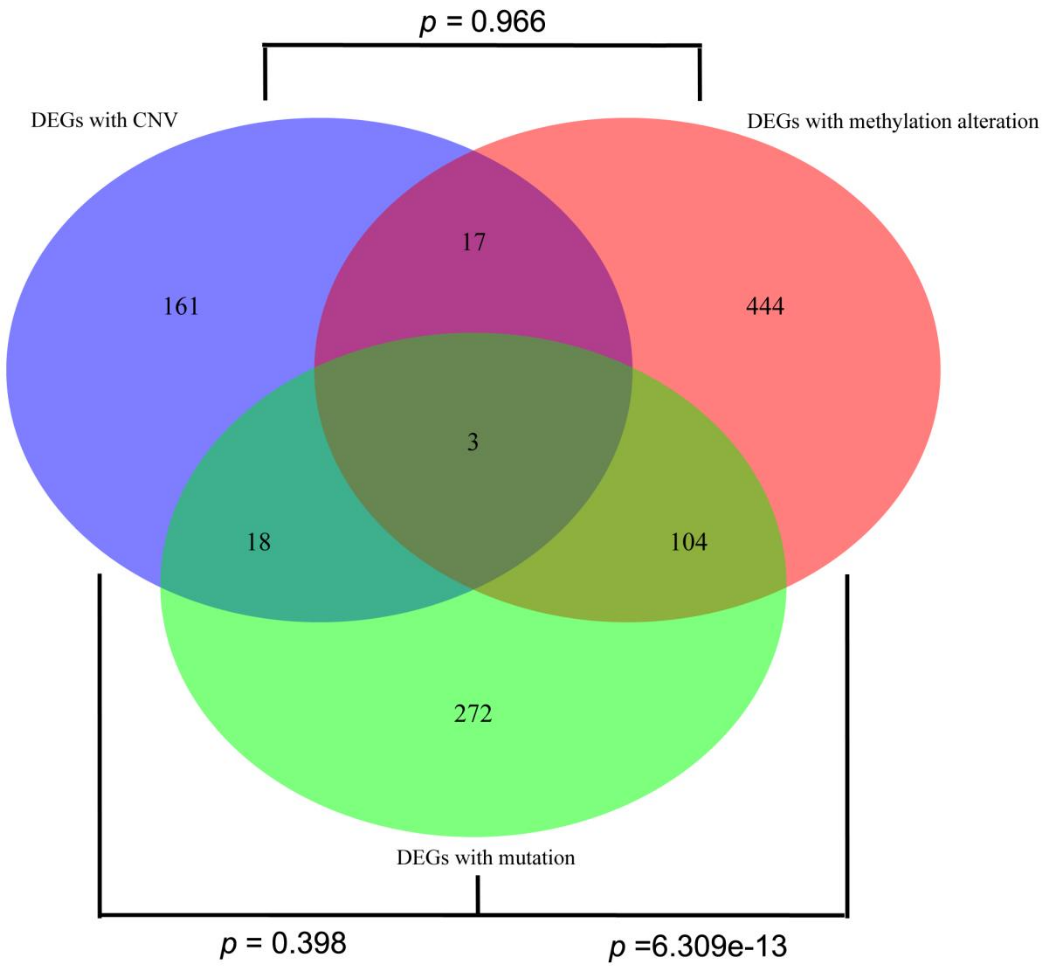

Supplementary Figure S2. Overlapping among three candidate gene groups. The statistical significance of overlapping was calculated via hypergeometric distribution, and the corresponding *p* value was presented between each two gene groups.

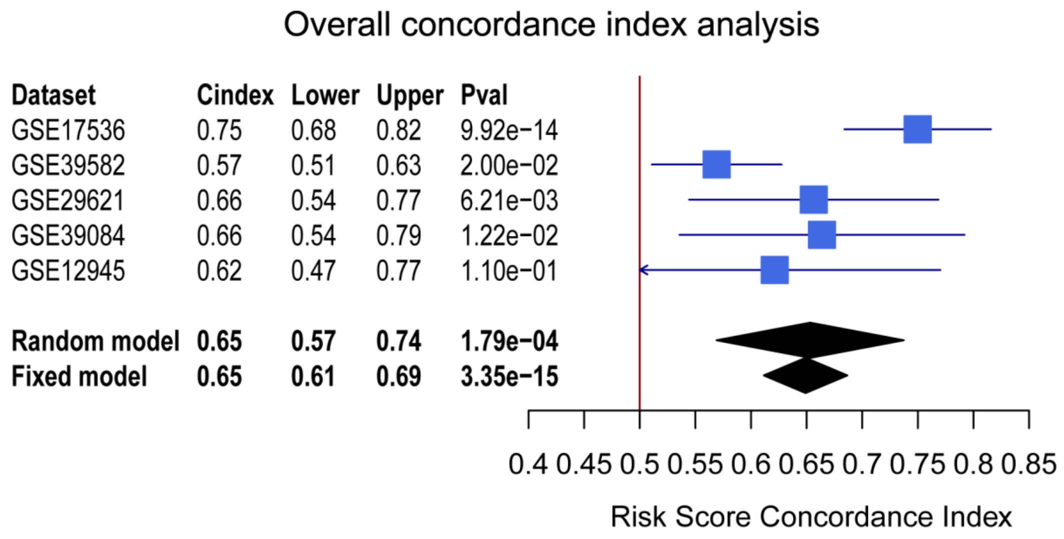

Supplementary Figure S3. Forest plot of overall concordance index analysis to evaluate the predictive ability of the 37-gene signature.
